# Practical Manual of the Diseases of the Heart and Great Vessels

**Published:** 1844-01

**Authors:** 


Art. VI.—*Practical Manual of the Diseases of the Heart and Great Vessels. A work intended to facilitate and extend the study of these Diseases.* By F. A. Aran, Interne of the First Class of the Hôtel-Dieu, Laureat of the Hospitals, formerly pupil of the Practical School of Medicine, &c. Translated from the French; by Wm. A. Harris, M.D. Philadelphia. Ed. Barrington & Geo. D. Haswell. 1843. pp. 296, 12mo.

The aim of this little work is a most praiseworthy one. That class of affections, the study of which it is intended to facilitate and extend, has hitherto been much neglected by the profession. We hope this Manual, comprising, as it does, a summary of what has been published in more elaborate works, will be extensively read, and that the study of it may lead to a more correct appreciation of the character of these diseases. A scepticism has prevailed, but too generally, as to the possibility of recognising and curing the complaints of the heart, and hence has arisen the indifference with which they are regarded by many physicians. This incredulity has been strengthened by the differences of opinion among authors respecting such affections. The author of the volume before us, familiar with the treatises of Senac, Corvisart, Laennec, Bertin, Bouillaud, Gendrin, and Hope, and guided by them in his researches, has brought the observations of these authors to the test of clinical experience. "We have not," he says, "adopted servilely the opinions of any one. We make no pretensions to novelty; but we have compared, and, as well as we have been able, have verified, for ourselves, the observations of others." We believe we shall be rendering our readers an acceptable service by presenting a condensed account of the matter of the Manual.

The author introduces his subject by the very just remark, that a study of the phenomena and all the conditions appertaining to the healthy state of organs is indispensable, in order to understand the various modifications they undergo in disease. Unless we are acquainted with the phenomena incident to the normal state, it is plain that we cannot judge with any degree of accuracy respecting the nature of morbid changes. The good practical rule, therefore, laid down by writers, ought always to be observed, "of uniformly investigating with care, the condition of each individual organ of importance, in every disease of whatever kind and character." By such a course of procedure practitioners would at once recognize any departure of the heart from its normal action, and acquire, at length, the tact in diagnosis upon which must rest any success in the treatment of cardiac diseases.

The first part of the Manual is devoted to the considera- of the anatomy of the heart, the physiology of its beats and sounds, the pathological physiology of the same, the arterial pulsations in their physiological and pathological state, and the sounds of the arteries in their healthy and morbid state. We shall not attempt any abstract of this portion of the work, but hasten to give an analysis of that part which treats of the *pathology of the heart.* Having presented the local symptoms of these diseases in the first part, he thus details the

"*General Symptoms:*—The diseases of the heart exert a very marked influence over the whole economy; nor is it in a narrow and circumsbribed circle that these morbid reactions are produced; but, on the contrary, how numerous are the sympathies which the central organ of the circulation creates in the rest of the organism! It is in consequence of their multiplicity, and the difficulty of referring them to one perfectly settled cause, that we have decided to study the general symptoms in a purely analytical order:

1st. *Congestions.*—The embarrassment of the circulation is marked in the skin, and principally in the face; sometimes by a violet and livid discoloration, general or local; sometimes by a livid paleness, which has a frightful appearance. It is not an uncommon event, to see the face of a blue color in its whole extent; but it is much more rare to see all the body present this color, except in certain congenital diseases of the heart. The engorgement of the venous system is one of the extraordinary phenomena pertaining to diseases of this organ; but in some circumstances, true beats, of more or less regularity, are observed in the veins near the heart (as the internal and external jugular). As this phenomenon constitutes a very valuable sign in cardiac diseases, it is important to distinguish the swelling of the veins from congestion, from the venous beats, or the swelling from regurgitation of these vessels. It is for the purpose of establishing this distinction, that M. Gendrin proposed a very ingenious process, which consists in making compression on the part of the jugular vein, most distant from the heart. If, in this case, the vessel remains empty and collapsed, the swelling of the veins does not depend on the regurgitation of the blood of the heart into the veins, but from an insufficient depletion of these vessels; if on the contrary, after having forced the blood from below upwards into the external jugular vein, and placed a finger on the most inferior part of this vessel, the blood is seen to reascend in the vein, and there produce pulsations, it may be affirmed, that the swelling of the veins depends on the reflux of the blood which distends the heart. The swelling of the veins with beats does not belong, therefore, to congestion, but rather to regurgitation. These beats, appreciable only to the sight, are perceived especially in the superficial veins of the neck; and sometimes in the internal jugular. They may be simple or double: when *simple*, they are always synchronous with the systole of the heart; when *double*, they are composed of an undulatory tremor which precedes the systole, and by a true beat, synchronous with the systole of the ventricles. The simple pulsations of the veins (*systolic*) may depend, either on a deficiency of the tricuspid valve, or on a considerable dilatation, with hypertrophy of the right ventricle; in this last case, the phenomenon is due to a regurgitation of the blood placed between two segments of the now thin and badly supported tricuspid valve, the motion of which is transmitted to the blood of the auricle and superior veins, (Hope). The double pulsations depend, first, on hypertrophy of the right auricle; second, on deficiency of the tricuspid valve, or on dilatation with hypertrophy of the right ventricle. These double beats are at their maximum where there is contraction with deficiency of the right auriculo-ventricular orifice. In the mucous membranes, the congestion is not less evident; and hence we see it supervening in the intestinal tube, and characterized by a punctuated redness, or by flakes of mucus in the stomach and intestines. This congestion, which is always dangerous, is announced by anorexia, eructations, severe pains extending through the abdomen, and later by serous diarrhœa, sometimes very abundant, and which is often accompanied by very prominent hemorrhoidal tumors.

"The functional vascular apparatus of the lungs (which is so immediately connected with the action of the heart) is in some measure the first to suffer from alteration of this organ. Hence, it is in the lungs that the first general phenomena make their appearance when they are dependent on embarrassments of the venous circulation; the first of these phenomena is the œdema of the lungs, which is indicated by a dry cough, considerable dyspnœa, evident diminution of resonance at the base of the lung behind, and the presence at this point of a crepitant moist râle with distinct bubbles; and, anatomically, by an increase in the consistence of the pulmonary parenchyma, which is like paste, its pale and greyish color, and in its general infiltration with a yellowish, frothy serosity. It is not uncommon to find, at a more advanced period in the diseases of the heart, the lung tumefied, with an increase of density in certain points, and infiltrated with a yellowish-red serosity, which flows with difficulty. These cases of œdema may be easily confounded with pneumonia; for independently of considerable dulness, there is found either a moist crepitant râle, or a true tubular sound. It is true, that the absence of the rusty sputa, of the symptomatic fever, and of the pain in the chest, prevent us from confounding these two diseases; but these cases are not less troublesome. The pulmonary œdema may manifest itself, and disappear many times; it often alternates with a phenomenon which we shall soon study under the name of colliquative diuresis, and has its maximum of intensity in the cardiac diseases, in which the circulation is found most embarrassed, as in the case of deficiency of the mitral and tricuspid valves. The pulmonary œdema unformily precedes the hydropic effusions which take place in the serous cavities.

"Pulmonary œdema precedes also that state of sanguineous engorgement, in which this organ takes on a reddish-brown color, loses its elasticity, and furnishes after incision a considerable quantity of blood of a violet-brown color. This state of congestion of the lung, which is generally accompanied by hemorrhage, is characterized by extreme dyspnœa, with partial dulness, and by the moist crepitant râle, as in œdema.

"The liver, which is the centre of all the venous system of the vena porta, and which empties itself by large outlets into the vena cava inferior, suffers more than any other organ from obstacles which oppose the return of venous blood. This congestion of the hepatic organ is made known by considerable tumefaction. On one side, it extends inferiorly below the border of the false ribs on the right, and it descends in front to the level of the umbilicus; it forms a shining tumor, and most often yielding to pressure, which preserves entirely the form of the liver. In another direction, this organ ascends by its convex face, which is rendered evident by auscultation and percussion. The swelling of the liver adds often to the dyspnœa, and it announces almost always incipient ascites, especially when the inferior extremities are in a state of œdema, and there is meteorism of the abdomen. It is never complicated with jaundice, unless the liver be the seat of a phlegmasia: in severe cases, it is accompanied by meteorism, vomitings, diarrhœa, and swelling of the hemorrhoidal veins, with or without hemorrhage. These congestions of the liver are evidently caused by those diseases in which the return of the venous blood is most impeded; thus, they are most often seen in diseases of the auriculo-ventricular orifices, and especially of the left. They are produced more rapidly in proportion as the obstacle is situated near the venous circulation; if it is in the right ventricle, the congestion will happen much sooner than if it was in the left ventricle. Hepatic congestions are most often accompanied with congestion of the lung, since it is through the medium of this organ that the venous circulation becomes arrested. We have already spoken of the congestions which take place in the intestines, and which succeed those of the liver. It is easy to comprehend how this congestion may take place in the spleen, and finally, by degrees, reach, not only all the organs which contribute to form the branches of the vena porta, but also all those which pour their blood into the inferior vena cava. The congestion of these last organs is indicated most commonly by a particular circumstance, noticed for the first time by M. Gendrin, and which consists in the habitual excretion of a quantity of urine, much more abundant than the amount of liquids taken in. It is generally during the night that this excretion is principally abundant; the urine is white, watery, and almost always without sediment; it contains no albumen; its quantity varies with the stage of the disease; but augments as the latter advances. The *colliquative diuresis* is not always constant; when it does exist, the dyspnœa is generally diminished. The diuresis almost always precedes the anasarca, and is caused by obstacles which are situated at the orifices, and particularly at the auriculo-ventricular ones. It is, however, observed, in some cachexies, in chlorosis, for example.

"The brain is not protected from the congestions which take place in the other organs; the connection which exists between the cerebral affections and the diseases of the central organ of the circulation has always been noticed. Congestion under these circumstances is announced by the following symptoms: dull and continued headache, weight in the head, inclination to sleep, injection of the capillaries of the face and conjunctiva; at a later period succeeds a comatose state, and the symptoms of a paralysis more or less extensive.

"2. *Hemorrhages.*—There is only one step from congestion to hemorrhage. Thus, we see patients who, after having presented, for a long time, symptoms indicative of congestion of the brain, are suddenly attacked with all the symptoms which point out a cerebral hemorrhage, viz: sudden loss of consciousness, circumscribed paralysis, &c. These cerebral hemorrhages and the encephalic sanguineous congestion which precedes them, have been generally considered as the immediate effect of the excessive impulse given to the column of arterial blood by the left ventricle of the hypertrophied heart. We shall examine, under the article *Hypertrophy*, whether this opinion be well founded.

"It is not only in the brain, but much oftener in the lungs, that hemorrhages are observed; as we can readily understand by a knowledge of the situation of the lung. This organ, placed between the right and left side of the heart, receives continually, through the pulmonary artery, the blood which it is to transmit to the left auricle by means of the pulmonary veins; but if any material obstacle exists in one of the orifices of the left ventricle, and especially in the left auriculo-ventricular orifice, the blood which at first stagnates in the left auricle, accumulates gradually in the pulmonary veins which are distended, then in the delicate vessels of the lung. These last either allow the blood to transude on their surface (*hemorrhage by exhalation*), or else are torn to give passage to this liquid (*pulmonary apoplexy*). Of these two forms of hemorrhage, the most common is certainly the pulmonary apoplexy (*pneumo-hemorrhage*). This affection, characterised anatomically by the sanguineous infiltration of a greater or less number of lobules, and by their conversion into black, homogenous tissue, is commonally announced by intense dyspnœa, more or less dull pain in the side, palpitations, expectoration, of a brown, and even black blood, dulness on percussion, and by the moist crepitant râle more or less mixed with the tubular sound. In general, pneumo-hemorrhages only supervene in the first periods of the disease of the heart, when the organism is not much deteriorated, that is, in persons who have not had œdema, and who still present all the appearances of good health. At a later period, when the contractility of the heart is enfeebled, and the pulmonary vessels become by degrees habituated to this sanguineous congestion, these hemorrhages become rare.

"Hemorrhages of the liver, and of the gastro-intestinal tube, ordinarily take place at a more advanced period in the diseases of the heart. These, when they are very abundant, often bring temporary relief for a certain length of time; finally, in the last stages of the diseases of the heart, there are sometimes observed sanguineous effusions, in the tissue of the eyelids, for example, and in the sub-cutaneous cellular tissue of the face. These sanguineous congestions may be increased in an alarming manner, and may even take place in the heart itself (Gendrin).

"*Serous Infiltration.*—The serous infiltrations are seen sooner or later in almost all severe diseases of the heart. They first take place in the cellular tissue (*œdema*, *anasarca*), and later in the serous cavities (*dropsy*); commencing always in parts of circumscribed bodies, especially in the inferior extremities. At first the œdema only supervenes at the end of the day, and disappears on lying down; it induces no change of color in the skin, and only a feeling of oppression; it preserves a long time the impression of the finger; but in proportion as the infiltration progresses and advances to the trunk, it no longer disappears completely on lying down. The œdema next occupies the cellular tissue of the parietes of the thoracic and abdominal cavities; and it may even become general. When it shows itself in the superior extremities, it is especially the right which is the most affected. We have for a long time sought for the reason of this. May it not be owing to the habit which patients have of lying on the right side, and consequently rendering it lower?

"To this general anasarca is added very often considerable dyspnœa, proceeding from the pulmonary œdema, which is a constant attendant, as well as from the infiltration of the thoracic parietes, which concurs to embarrass considerably the movement of the muscles. The anasarca is always accompanied by a considerable deterioration of the functions. The organs which only receive very poor blood fall into a state of inertia; the muscles become atrophied; the skin and all the mucous membranes become dry; the renal secretion diminishes; so that, when we have succeeded in causing the anasarca to disappear, we always find the strength of the patient so enfeebled, and the general complications so aggravated, that there is little hope of re-establishing the healthy organism. It is only when the anasarca has increased to a very great extent, that infiltrations take place in the serous cavities; they are at first effused into the peritoneal cavity, then into that of the pleura and pericardium, and sometimes even into that of the arachnoid.

"These serous effusions add to the gravity of the primary disease: the ascites renders the respiration more difficult, increases the embarrassment of the circulation in the abdominal vessels, induces vomiting and constipation; the hydrothorax diminishes the extent of the pulmonary surface, contracts or diminishes the space for hematosis, and increases considerably the dyspnœa; the effusion into the pericardium augments the irregularity in the movements of the heart; and the serous effusion into the arachnoid, which throws the patient in a state of profound coma, often puts an end to his agonising sufferings.

"4. *Gangrene of the Extremities.*—Gangrene of the extremities is sometimes seen in individuals of an advanced age, as a complication of the diseases of the heart. It only happens at a very advanced stage of the disease, when the whole organism is much deteriorated; that is, when the anasarca is constantly reproduced, and when the patient is much emaciated and complexion deteriorated; it most often happens after scarifications of the inferior extremities; the presence of œdema is always one of the principal causes. Sphacelus commences ordinarily in the skin of the toes or fingers, which are covered with livid spots, or in the lips of the scarifications, which become black; at the same time the limb is the seat of acute pains, with a feeling of coldness; the spots increase in extent; phlyctenæ are formed, and the sensibility is extinguished in the points where the epidermis is raised. These spots rapidly ascend to the trunk in increased numbers, and below them the skin is always mortified. In proportion as the disease extends superiorly, the pulsations of the artery in the limb affected cease to be perceived, and the arterial tube is converted into a cord filled with coagulated blood. The veins of the limb become nearly effaced, and are only recognized by black lines which furrow the surface of the limb; at the same time, the beats of the heart grow feebler; then follows adynamia, wild delirium, eschars on the sacrum and great trochanters; finally, the patient dies comatose seven or eight days after the invasion of the sphacelus. Such is the gangrene which is a consequence of diseases of the heart. Gangrene of the extremities may also be caused by diseases of the arterial tubes; it is this species that the surgeons have described under the name of *senile gangrene;* but this is not the place to speak of it.

"5. *Dyspnœa.*—The serous and sanguineous congestions which almost always have their seat in the lungs, and the difficulty of hematosis, which is a consequence of them, produce always some difficulty in the respiratory functions. The patients do not at first take notice of their dyspnœa; afterwards they find that they are much out of breath when they take exercise. It is not rare to find, in the working classes, and in children, individuals who present the physical signs of severe diseases of the heart, and yet they say that they have never felt dyspnœa. The reason is, that these individuals pay little attention to their sensations, and that, as the dyspnœa comes on gradually, they become accustomed to it, and never think that they are afflicted with any disease.

"The dyspnœa increases continually, and often imposes on the patients the necessity of preserving the most absolute repose. This affection is not always felt to the same degree. When at rest, the patients suffer little or none; but deviations in diet, moral emotions, and corporeal labor increase its intensity. It has not always the same causes: sometimes it is due to the sanguineous or serous congestion of the bronchial mucous membrane; sometimes to a chronic bronchitis, or to a very marked pulmonary emphysema. But the fixed cause of this dyspnœa is always found in the insufficient oxygenation of the blood; either because the air only penetrates the aërial vesicles and bronchial ramifications in a very small quantity; or because the permeable state of the mucous membranes prevents the blood from being sufficiently exposed to the action of the air; or, finally, because the blood does not flow in sufficient quantity into the pulmonary vessels.

"Dyspnœa is generally one of the first signs of diseases of the heart; sometimes it is habitual, and sometimes it is paroxysmal. Most commonly habitual, it also presents exacerbations. It is this last which constitutes one of the great varieties of asthma, that is called *cardiac asthma.*

"*Cardiac asthma* presents various characters; sometimes it is *humid*, for example, when the lungs are in a permanent state of engorgement, as happens in the case of contraction of the left auriculo-ventricular orifice; it is *dry* when the engorgement of the lung is only temporary, as happens in simple hypertrophy; finally, it may be *convulsive.*

Sometimes the attacks of asthma come on suddenly; sometimes (in severe cases, for example), they are announced by some precursory troubles, as feeling of weight in the epigastric region, distension of the abdomen, pain, oppression, constriction in the forehead with some beating, and an indefinable sensation of oppression and anxiety in the præcordial region. At other times, the invalid is uneasy, irritable, morose, and it may be apathetic or melancholy. Whatever was his previous state, the attack begins in the following manner: the patient, if he is lying down, wakes up suddenly with violent palpitations, with an agonizing sensation of oppression in the præcordial region, and a considerable tightness in the chest, as if it was forced in a vice: he raises himself upon his seat and demands instantly air: the respiration is noisy, and is accompanied with violent efforts of all the respiratory muscles; the inspirations are long, the expirations short and imperfect; the whole external surface feels cold; the face is pale or livid; the pulse frequent, small, feeble, often irregular and intermittent. In proportion as the attack loses its intensity, the oppression and tightness of the chest diminish, the respiration becomes less frequent, less loud, and less labored; the pulse slower, fuller, and more regular; the patient retains only a slight constriction of the chest, and a slightly noisy respiration. Finally, the attack terminates by a very abundant expectoration of a viscid liquid, which is transparent, or by a considerable diaphoresis; sometimes by a copious evacuation of pale and clear urine. As long as these evacuations, which constitute the crisis of the attack of cardiac asthma, and which announce the disengorgement of the pulmonary system, do not take place, it is to be feared that the attack will soon reappear. In general, the attacks of asthma are more complete if the obstacle to the circulation is great, and the pulmonary congestion continues long. Thus, in the case of disease of the valves with considerable dilatation, the attacks often last for five or six hours. In proportion as the organic disease of the heart progresses, the attacks of asthma, which at first only returned at distant intervals, come nearer to each other; the respiration, always short, is now quick and laborious from the least exercise, or from the least moral emotion; finally, in the last period of this disease, the cardiac asthma is persistent. Unable to remain in the horizontal position, the patients remain for weeks and whole months in almost a vertical position, and sustained by pillows, their body bent forward, their elbows placed on their knees, their eyes widely open and staring; their eyebrows raised, and their nostrils dilated. Their features are dark and haggard, and their head is drawn backwards at each inspiration; they cast around them a mingled look of horror and entreaty; sometimes praying with an almost inaudable voice, and with plaintive cries, or in feeling and broken accents, for the assistance which has been freely administered to them in vain: sometimes accusing the impotency of medicine; sometimes, finally, in the agony of despair, letting their head fall on their chest, and murmuring a fervent request that death would put an end to their sufferings. For some hours, sometimes only for some minutes, they experience a delightful interval of calmness. They flatter themselves with the hope that their distress is passed, and their cure is at hand; but this hope soon vanishes; the short sleep which they enjoy is interrupted by the horrors of an unfortunate dream; they raise themselves up, uttering piteous cries. Finally, the respiratory muscles, overcome by the efforts that the instinct of self-preservation has imposed upon them for so long a time, participate in the general exhaustion, and refuse to fulfil their functions. Then the patients expire in the midst of a last inspiration, (which is most rarely the case), or fall exhausted on their bed; the respiration in this case is short, raucous, almost absolutely diaphragmatic; the skin becomes violet, the pulse almost imperceptible, and the patients fall into a state of comatose asphyxia. This comatose state may last some hours; we have seen it last for three days.

"6. *Nervous Disorders.*—We include under this head all those disorders of innervation which have an influence either over the general or special sensibility of certain organs and tissues. Amongst these disorders, we notice especially acute pains which the patient experiences in the epigastric region when he partakes of the indulgences of the table; and which are accompanied by eructation in all cases. We must also notice those pains which radiate from the epigastric region towards the left shoulder, and which are accompanied by more or less painful numbness in the superior extremity of the same side (*angina pectoris.*) These pains may not at first descend below the deltoid muscle, but they soon extend along the internal part of the arm to the elbow, sometimes even to the extremities of the fingers, following the course of the cubital nerve. Their duration is almost always short: when they are prolonged, they give rise to a suffocating dyspnœa, to violent palpitations, to syncope, and even to convulsions.

Generally the attack is induced by all the causes which excite the action of the heart, as walking, running, the pleasures of the table; it seldom lasts more than ten minutes, and still more rarely continues for half an hour or an hour: in this last case the danger is imminent. *Angina pectoris* is only present in very advanced and severe disease of the heart. It is at its maximum in cases where there are osseous or cartilaginous and steatomatous degenerations of the heart and large vessels, or where there is hypertrophy and considerable dilatation of the organ with or without softening of its parietes."

We copy next his remarks on the *mode of investigation in the diseases of the heart:*

"One of the causes which has most retarded the progress of the pathology of the heart, is certainly the want of method in the investigation of the morbid changes of this organ. The diseases of the heart are composed of a number of phenomena, all having a particular signification. It is necessary, therefore, to proceed to their examination in a methodical manner, in order that none of them may be omitted. This is the mode which appears to us most proper; it is similar to that which M. Gendrin has followed.

"To examine properly patients affected with diseases of the heart, it is necessary to place them in an almost horizontal position; to take away all clothing that may either embarrass the motions of the chest, or produce artificial sounds by friction. It is preferable to examine them in the morning, after sleep. It is necessary to uncover all the præcordial region in men, and place only very fine linen on the anterior surface of the chest in women.

"We must first find out, by the sight and touch, that there is no anomalous tumor seated in the præcordial region, and that the thoracic cavity presents no kind of deformity; then applying the hand over the præcordial region, we must try to find exactly the seat of the apex of the heart, and its distance from the axis of the sternum. At the same time we must judge, by the hand applied over the heart, of the force, the extent, the number, the duration, and the regularity of the beats or impulse of this organ. A knowledge of the position of the apex of the heart is one of the most important elements in diagnosis, since it permits us to determine the length of the organ, by measuring the distance which separates this point of the thorax from the intercostal space where we have said that the base of the heart is invariably fixed.

"We then make percussion over the præcordial region, by striking lightly, with one or two fingers of the right hand on one of the left hand, applied immediately over the intercostal spaces.

"We thus estimate the extent and degree of dulness, and at the same time also the position of the anterior border of the lungs, the volume of the heart, and that of the pericardium.

"We pass, next, to the auscultation of the præcordial region. In this examination, the ear is most often made use of; although when we wish to limit the surface to be auscultated,or when the structure of the parts opposes the immediate application of the ear, the stethoscope is used. The form of this instrument is not of much consequence, provided that its ligneous fibres be continuous from one extremity of the cylinder to the other. The rules to follow for the application of the stethoscope in the diseases of the heart, are the same as those for diseases of the chest; it is only necessary to take out the top, which Laennec added to it. The first thing to do in the auscultation of the heart, is to isolate it as much as possible from the sounds which take place in the chest. When these are very intense, it is necessary to tell the patient to hold his breath for a few minutes: applying then the ear over the place where the apex of the heart is seated, we hear sounds, and can determine the modifications which they experience in their force, extent, tone, duration, and rhythm, as well as the modifying anomalous sounds, by referring them to the two functional acts and to the two sounds of the heart. From the apex of this organ we extend the examination to the left, at first transversely, then ascending to the border of the pectoralis major, and descending to the inferior limits of the left hypocondrium (as it is in this space that we find the maximum of the sounds which are produced in the left ventricle), in order to examine whether the normal or abnormal sounds increase or diminish in going towards this point, whether any anomalous sounds are added, taking into account the respiratory phenomena, and the transmission of sounds which may take place in consequence of a disease of the left lung. We then go to the right, and within the space from the apex to the sternum (maximum point of the sounds which take place in the right ventricle), examine the modifications in the phenomena which are perceived at the apex. In this last examination, we must take care, and not be misled by one cause of error. The anomalous sounds of the aorta are often perceived at this point, and may be mistaken for anomalous cardiac sounds. To avoid this error it is sufficient to remark—1st. Their constantly synchronous character with the systole of the heart, which belongs to the aortic murmurs. 2d. Their extension over the whole course of the sternum, often even under the linea alba; whilst the murmurs of the right side of the heart may be systolic or diastolic, and never are prolonged to this extent.

"Setting out again from the apex of the heart, we ascend from below upwards, over the course of this organ to its base. In our progress, the modifications which the normal sounds undergo, and the changes taking place in the anomalous ones as heard at the apex, are rendered evident. We make the same examination when we have reached the base, and we note in the course of the pulmonary artery, then of that of the aorta and its ramifications, what modifications the normal or abnormal sounds undergo; and whether new ones are developed. We follow these sounds, particularly in the brachio-cephalic trunk, the subclavian, the external primitive carotid, sometimes the internal, by applying the stethoscope on the base of the orbit, as M. Gendrin does. We then follow the course of the aorta in the abdomen, and of its iliac and crural branches.

"The investigation to be made respecting the arterial circulation demands much care. Thus, when we examine the arteries which are enclosed in the thoracic cavities, we must, as much as possible, isolate the sounds of these vessels from the respiratory sounds, and especially from the normal and abnormal ones of the heart. In the abdomen, we must also distinguish the arterial sounds: 1st, from those of the heart, which latter sometimes extend even to the abdomen; 2d, from intestinal borborygmi; 3d, from the muscular sound which is developed in the abdominal parietes when they are not sufficiently relaxed, and when they contract under the pressure of the stethoscope. When, on the contrary, we examine arteries which are only covered by soft parts, there are some especial precautions to take. We must not press too much with the stethoscope, nor relax the parts which cover the artery. When we auscult the arteries of the neck, the patient should have his head supported by a pillow, and a little stretched, but the face turned forwards. Next we must examine by making the parts tense: in the neck we turn the head to the side opposite to that on which we examine, raise the chin, &c., to ascertain whether the modifications of the arterial sounds are similar on both sides. At the same time we recognise by the touch the state of these arteries; we study the effects of pressure on them, the modifications of the beats, in their force, frequency, and rhythm. Resuming the exploration of the trunk behind, we ascertain, over the course of the lateral parts of the spine, whether the sounds of the heart and great vessels are heard in these points, and what are the modifications which they have undergone.

"Finally, we conclude by noting the color of the face, the state of the cutaneous capillaries, and of the different mucous apertures, the state of the pulse in different parts of the body, the condition of the viscera; at first of the lungs, then of the abdominal contents: we carefully note, also, the state of the abdomen, the presence of infiltrations, or serous effusions, &c.

The author speaks of the affections of the heart under three heads: *inflammatory diseases; organic diseases; and nervous diseases.* Under the first are embraced pericarditis, carditis, endocarditis, and arteritis; under the second, hypertrophy, atrophy, and dilatation of the heart; partial dilatation or true aneurism of the heart, softening, induration, excessive adipose deposit and fatty degenerations of that organ; osseous, cartilaginous, and other accidental productions in the muscular tissue of the heart, and on the pericardium; diseases of the valves and orifices of the heart, aneurism of the aorta, of the pulmonary artery, and malformations of the heart; and under the third, palpitations and syncope, to which an appendix is added on hydro-pericardium, hemo-pericardium, and pneumo-pericadium, and on polypi, or sanguineous concretions of the heart.

Passing over the symptoms and causes of pericarditis, we quote the remarks of our author on its

"*Diagnosis.*—When the pain is seated in the præcordial region, and is aggravated by pressure; when there is increased action of the heart, and a violent febrile reaction, the existence of pericarditis may be suspected. If, besides, the pulse is feeble, trembling, irregular, &c., without our being able to detect the cause; if we observe all the signs of embarrassment in the circulation: and finally, if there is dulness on percussion, and obscurity in the sounds of the heart, there can be no doubt of the existence of pericarditis. But it may so happen, that there is neither pain nor considerable change in the pulse, no difficulty of respiration, no dulness appreciable by percussion, nor feebleness of the sounds of the heart. If, however, the beatings of the heart be violent and bounding without any manifest cause; if there be anxiety and a high febrile reaction; and finally, if we recognize the friction sound of the pericardium, there is little danger of our being mistaken in diagnosticating pericarditis. As we have already seen, the diagnosis of acute pericarditis, in which we are assisted not only by general symptoms, but also by the results of percussion and auscultation, presents great precision. The most difficult cases are those complicated with diseases of the brain and other organs, and which divert the attention that ought to be given to the examination of the heart. We may say plainly, if it was a general custom to place the hand over the præcordial region, as often as it is placed on the pulse in all inflammatory diseases; if the heart was examined with as much care by percussion and auscultation as the lungs are, it would be a rare occurrence to mistake a pericarditis. This pretended diversity and variability of the symptoms, in which most of the pathologists now place the cause of obscurity of pericarditis, are still not without value in throwing light on the disease. Although they may cause temporary hesitation in the mind of the physician, yet they furnish valuable indications. These multiplied variations are in a direct proportion to the nature and progress of the anatomical changes of structure, and consequently to the progress and the stage of the disease. Generally, it is in the difference of the quality and quantity of the effusion that we must seek the difference in the aspect of the symptoms. If the effusion consists principally of plastic lymph; if the serosity has been rapidly absorbed; in a word, if the adhesions of the pericardium are rapidly formed, the movements of the heart present, during the whole period of the disease, the same vigor and regularity as at the beginning; the pulse retains its characteristic force, hardness, regularity; the patient is less constrained in his postures, and life may continue for several weeks, even though the inflammation has not been removed. This mild form of the disease is observed still better when, instead of producing adhesions of the pericardium, it rapidly progresses to the state of resolution. If, on the contrary, the serous effusion be not absorbed, the movements of the heart are impeded by the mechanical compression of the liquid on this organ; and hence ensue a small, intermitting pulse, and all the symptoms which announce embarrassment of the cardiac circulation, such as feebleness, dyspnœa, anxiety, coldness of the extremities, lividity of the integuments, &c. If the serous effusion be abundant at the beginning, all these symptoms manifest themselves early in the disease; but as, in general, two or three days are required for a considerable accumulation of the liquid, to those which we have just described, suddenly succeed the symptoms indicating a strong and regular action of the heart. It must, however, be confessed, that there are certain cases of pericarditis, in which all the symptoms of difficulty of the circulation are observed, and there is still very little effusion of liquid. In most of these, there are very thick false membranes which act on the heart, in the same manner as the effused liquid; in other cases, it is impossible to explain the occurrence of the accidents otherwise than by supposing complications of other cardiac affections (carditis, endocarditis, polypi of the heart), or of the pulmonary organs (pleurisy or pneumonia), or by an increase of irritability. According to M. Bouillaud, the great differences observable among the general symptoms of pericarditis depend rather on a violent pleuritic or pleuro-pneumonic complication, than on the pericarditis itself. No doubt these complications aggravate the symptoms, and impart to them the highest degree of intensity; but this opinion of M. Bouillaud cannot be admitted in an absolute manner, since we often see pericarditis with dangerous symptoms, without any trace of complication. We have now considered the general diagnosis of pericarditis; there yet remains to be noticed the differential diagnosis.

"Pericarditis can only be confounded with inflammation of some one of the thoracic viscera, and especially of the pleura. These complications which, from the time of Corvisart, were one of the greatest obstacles to the diagnosis of pericarditis, no longer embarrass us, since the introduction of auscultation. It is well known that pleurisy, independently of the general signs, is recognized by the following physical ones: dulness on percussion, commencing at first in the posterior and inferior part of the chest, and only extending forwards at a later period in the disease; diminution or complete absence of the vesicular murmur in all the points which are the seat of the dulness; bronchial respiration, and œgophony, when there is only a small quantity of liquid. Pneumonia, besides these general ones, and in particular rusty and viscid sputa, is announced by the following signs: in the first stage, crepitant rhonchus, and commencing dulness on percussion; in the second stage, cessation of the crepitant rhonchus, and of the respiratory murmur; dulness evident on percussion; tubular sound and bronchophony; slight dilatation of the affected side, increase of the vibratory tremor of the voice. In fine, bronchitis is recognized by the presence of mucous, sibilant, and sonorous rales, and by the absence of anomalous dulness. If no one of these sounds be found, we are forced to the conclusion that the heart is the seat of the disease; but if, on the contrary, some of them are recognized, they should be compared with those which properly belong to the inflammations of the pulmonary organs, taking also into careful consideration the anomalous sounds which supervene in the heart, as well as the direction in which the dulness is perceived. After all, if we are in doubt, it would be proper to act as if this inflammation existed, adapting, at the same time, the treatment to the pulmonary complications as they arise; because it is much easier to subdue the diseases of the heart at their commencement, than when they have long had hold of this organ. There remains the differential diagnosis of endocarditis, which will be considered in the following chapter.

"The obscurity of the symptoms of chronic pericarditis, and especially of this disease at its commencement, renders its diagnosis more difficult than that of acute pericarditis. Notwithstanding, with the aid of auscultation, these difficulties in a great measure disappear. In short, if a patient, who had not had previously disease of the heart, presents all the symptoms of this disease, with general emaciation and hectic fever; if he refers the attack of the disease to a blow or fall on the chest, to acute articular rheumatism, or to an inflammation with pain in the præcordial region, we may suspect chronic pericarditis; if to all these symptoms be added dulness on percussion, or the pericardiac friction sound, no doubt can exist respecting the existence of this disease."

Nothing concerning the treatment of the disease is submitted which would be new to the American practitioner. An inflammatory affection of such a part imperiously calls for antiphlogistic measures.

General carditis, Aran thinks, is not so rare a malady as Laennec supposed it to be, and he is inclined, with Bouillaud and Professor Gross, to refer to inflammation of the heart, the softening as well as the induration frequently met with in that organ. He gives the following symptoms:

"The disease begins by a peculiar uneasiness of the præcordium, with slight pain in this region; palpitations, at first fugitive, and of slight intensity, soon followed by irregular movements; but, notwithstanding these symptoms, the sounds of the heart are not notably altered, except in their intensity; the dulness of the præcordial region being in its normal condition. Fever is soon lighted up; the pulse becomes strong, full, and resisting; there is difficulty of respiration, with orthopnœa, and extreme anxiety. To this reaction succeeds a period of depression; the sounds of the heart, before so violent, become intermittent; the difficulty of circulation is extreme; the face is flushed; the extremities are œdematous and cold; the dulness of the præcordial region increases in consequence of the congestion of the heart; the lungs are gorged; the pulse is feeble and fluttering; and the patient dies by a species of asyhyxia."

Partial carditis is characterised sometimes by softening or induration of the heart; sometimes by the existence of abscesses or ulcerations on one of its surfaces. Abscesses are more rare, according to our author, than ulcerations; and neither afford any sign by which they can be distinguished from other affections of that organ. Ruptures of the heart are owing most frequently to ulcerations; the other conditions which give rise to it are, ordinarily, softening or fatty degeneration. The author adds,

"These ruptures are most frequently produced by causes depending on violent efforts, paroxysms of anger, external violence, &c. They are announced by the following signs: pungent pain in the præcordial region; loud cries; great paleness; instinctive contraction of the extremities; syncope, though sometimes this affection comes on later; and the invalid, after having remained more or less time in this state, is seized with symptoms of reaction, which causes death in a few hours. According to many authors, in more fortunate cases, all the symptoms are observed to lose their severity; the respiration and circulation are established; the blood is absorbed; a cicatrix forms at the point of rupture, and a cure takes place in fifteen or twenty days. As ruptures of the heart kill almost instantaneously, it is always difficult to recognize the disease before the patient has died; however, when death does not take place so rapidly it may be suspected from the presence of the preceding signs, and of those which announce an effusion into the pericardium. Ruptures can only be confounded with sudden apoplexy, ruptures of the diaphragm, rupture of the large vessels of the chest, or with syncope. Apoplexy, whatever be its intensity, does not immediately stop the beatings of the heart, and these preserve their normal character; ruptures of the diaphragm do not kill in so sudden a manner; syncopes do not prevent the pulsations of the heart from being heard, and they cease often, in a certain space of time, by posture and stimulants, &c.; there remains a rupture of the large vessels, especially in the pericardium, which it is impossible to distinguish from rupture of the heart, otherwise than by the antecedent symptoms."

Endo-carditis is the name given to inflammation of the internal membrane of the heart—a disease mentioned by Corvisart, Laennec and others, but which has been investigated with peculiar ability by Bouillaud, who pointed out the coincidence between this affection and acute articular rheumatism. We again copy from the Manual our author's remarks on the diagnosis of the disease:

"As a general rule, when called to a patient presenting, 1st, a violent febrile reaction; 2d, an active impulse of the heart; and, 3d, an intracardiac murmur (blowing sound) which did not exist before, we may admit the existence of endocarditis.

"There is one point on which we insist; it is the differential diagnosis of pericarditis and endocarditis; independently of some differences in the general symptoms (as the greater intensity of pain in pericarditis, and the rapidity with which dangerous complications supervene), the differential diagnosis of these two diseases rests, in a great measure, on the dull and stifled character which the sounds of the heart present in pericarditis, and on the differences which exist between the friction sounds belonging to the pericardium, and the valvular murmurs. Now is the time to make known the differential characters of these two sounds:

| *Valvular Intra-cardiac Murmurs.* | *Friction Sounds.* |
|---|---|
| 1st. The valvular murmurs are always deeply seated and distant. | 1. The friction sounds are always superficial, and take place immediately beneath the ear. |
| 2. They are commonly simple. | 2. They are most often double. |
| 3. They generally have a soft character (that of the bellows sound), at least in acute endocarditis. | 3. They are rough, and when they coincide with the valvular murmurs, they are perceived, in some measure, through the medium of these last. |
| 4. The murmurs with the second sound, or diastolic, are very seldom rough. | 4. The diastolic sounds which present rough characters are probably friction sounds. |
| 5. The valvular murmurs once established, very slowly change their nature, and never their seat. | 5. The friction sounds rapidly and frequently change their nature and their seat. |

Of the treatment, the author remarks,

"The treatment of acute endocarditis ought to be neither less prompt nor less vigorous than that of pericarditis. We must not be misled by the apparent lightness of the symptoms, and we should never forget that an organic disease of the valves, whose development it is necessary to guard against, may supervene.

"When called to a case of endocarditis in the commencement, if the patient is well constituted and young, the antiphlogistic treatment should be rigorously made use of; repeated bleedings, after the method of M. Bouillaud, will be required, and produce the best results. When the patient is feeble, or very old; when he is affected with a former disease, or under the influence of some debilitating operation, it is proper to try only one general bleeding, and to renew local bleedings (leeches, cups); in some cases where endocarditis is only a very feeble element of the disease (as in certain acute articular rheumatisms), we may, when the general symptoms are not severe, limit ourselves to the treatment of the primitive affection. We have seen rheumatic endocarditis cured under the tratment directed against the rheumatism.

"When we have exhausted, without any beneficial result, all the antiphlogistic measures against endocarditis, such as general and local bleedings, diluting drinks, cataplasms, general baths, rest, and diet; or when fulness, or some particular conditions contraindicate their application, it is proper to have recourse to repeated blisters over the præcordial region. This is an excellent measure, and if we place on the denuded surface of the skin from 30 to 40 centigrammes of powdered digitalis, we may often notably diminish the feeling of uneasiness which the patient experiences in the region of the heart. It is almost always proper to add to the employment of blisters that of revulsives to the intestinal canal; and finally, when there is danger of the endocarditis passing into the chronic state, a deep cautery, which will suppurate and keep open, over the region of the heart, in order to exercise a powerful revulsion, at the same time that the patient is placed in a state of absolute rest, and submitted to cooling regimen. It is in this last case that we can have recourse to the treatment of Hamilton; that is, mercurial frictions over the region of the heart, and, internally, mercury combined with opium; digitalis and hyosciamus should also be used internally."

Hypertrophy of the heart, investigated by Corvisart under the name of active aneurism, is a disease of very frequent appearance, and the study of the causes which excite so formidable an affection is interesting. We are acquainted with a young man in whom it followed a fall from a tree, by which he sustained a violent shock: other causes are thus detailed:

"1st. *Physical Causes.*—Under this head are included all those which accelerate or retard the circulation, and augment the pressure of the blood on the heart. This class comprehends all violent and prolonged efforts (causes which accelerate the circulation); numerous alterations in the aorta, the valves of the heart, and the pericardium (as adhesions of the pericardium); certain pulmonary affections (emphysema, asthma, chronic catarrh); all abdominal diseases which cause the diaphragm to be forced upwards (the use of too tight corsets, &c.); all causes that embarrass the circulation, and increase the pressure which the blood exercises on the heart. 2d. *Physiological causes:* all moral emotions; all disorders of innervation that induce frequent palpitations. 3d. *Pathological causes:* inflammation, either of the heart itself, or of its two serous envelopes. So far we have considered the development of hypertrophy as subordinate to mechanical causes only; but it is now clearly shown, that chronic inflammation, whether original or consequent on acute inflammation, may cause hypertrophy of a muscular organ; this inference may very justly be drawn as regards the heart, or central organ of the circulation. Consequently, we are forced to admit, that when an organic lesion, the consequence of inflammation, has caused hypertrophy, the inflammation itself has contributed its share to produce this alteration.

*Diagnosis.*—Hypertrophy of the heart is distinguished, 1st, from *pericarditis*, by the nature of the dulness, the force and energy of the impulse, the absence of the friction sounds, and the reactional phenomena which belong to this disease; 2d, from *endocarditis*, by the increased extent of dulness, absence of the murmurs, and sympathetic phenomena, which accompany this disease; 3d, from *pleurisy with effusion*, which sometimes forces the heart under the sternum, by signs which announce this alteration in the seat of the organ, and the absence of that dulness in the posterior part of the thorax which belongs to pleurisy; 4th, from *pulmonary emphysema*, by the normal resonance of the whole chest, and dulness in the præcordial region.

"*Treatment.*—The first indication which is presented in hypertrophy, is to remove the known exciting causes, as corporeal exercise, intemperance, moral emotions, &c.; then it will be proper to have recourse to a measure (*sanguineous depletion*) which has been always recommended, but differently employed, in the treatment of this disease. Laennec after the example of Albertini and Valsalva, suggested very large bleedings, at intervals of two, four, or eight days; and a reduction gradually of the quantity of nourishment to fourteen ounces a day. This treatment should be continued for many months, at least; sometimes for several consecutive years. The end proposed by Laennec, is to unload the cavities of the heart, and to diminish the stimulating action of the blood and the active nutrition of this organ. The treatment of Albertini, applied rigorously, often counteracts its intention, and may hasten the death of the patient. We think that an equally fortunate result may be obtained by a mode which is more advantageous to the patient; which is, to draw six or eight ounces of blood, by venesection, every fifteen days or a month; according to the age or strength of the subject, and afterwards to have recourse to local bleedings, behind the ears, or over the præcordial region, if there be symptoms of cerebral or cardiac congestion. The nourishment should consist, during the first two or three months, of fresh fish, vegetables, and farinaceous articles, (only feeble persons may eat a little meat); the nourishment should be small in quantity, and the meals light, and so arranged as to satisfy the appetite, without overloading the stomach. The patient should have water, slightly flavored, for drink; those who are feeble may drink a little wine largely diluted. All kinds of exercise on foot should be prohibited; but riding in a carriage will be found useful. It will be well, during the first period of the disease, to join, to the use of bleedings, saline purgatives with the view of preventing constipation, and of producing a slight revulsion on the intestinal canal; the use of nitrate of potassa, digitalis, and other diuretics, will be indicated, even when dropsy has not appeared. Digitalis, independently of its diuretic action, has the property of rendering the beats of the heart slower, and of considerably diminishing their force: given in the dose of one grain to four grains of the leaves, or of ten to thirty drops of the tincture, it generally produces good effects. It is especially useful in simple hypertrophy, less so in hypertrophy with dilatation, and would be very pernicious when the dilatation has reached to a great extent. This medicine ought not to be continued during the whole period of the disease, as some persons practice. When we have obtained the effects which we expected, it is proper to suspend its use, in order to recur to it at a later period with advantage; if the digitalis be constantly employed, it will produce disorders of the digestive canal; cardialgia, nausea, vomiting, &c. There are some persons whose stomachs cannot tolerate it at all; in this case it should be used by enema, or the endermic method should be tried.

"When the dropsy is perfectly well marked, we have recourse to the most active diuretics, and to hydrogogues. (See *Treatment of the Diseases of the Valves.*)

"Under certain circumstances, where the irritation of the nervous system continues, notwithstanding the most jndicious measures, it will be found useful to administer some sedatives, as hyosciamus, one grain to four grains of the extract, the acetate or hydrochlorate of morphia in doses of from a tenth to a fifth of a grain, &c.

"The employment of iodine and mercurial frictions, as absorbents, has been proposed: but the results have not answered expectation.

"The treatment which we have just proposed, and which is very similar to that which Hope employed in his large practice, ought, in order to succeed, to be perseveringly continued for two or three years; and it will be necessary, for one or two years more, to take proper precautions in order to avoid relapses; but, although the treatment be thus prolonged, the patients easily submit to it, since it does not give them much trouble or distress."

Atrophy of the heart presents three variations:—1. *Simple atrophy*, the walls being attenuated without any notable change in their capacities, but with an appreciable diminution in size; 2. Atrophy with dilatation; 3. Atrophy with contraction. It is characterised by signs the opposite to those of hypertrophy—they are as follows:

"*Local Symptoms.*—Beats of the heart, short and feeble; impulse hardly appreciable; sensible diminution in the dulness on percussion; extreme feebleness of the sounds of the heart, which are slow and regular.

"*General Symptoms.*—Atrophy often exists for a long time, without being accompanied by general symptoms; individuals, who are affected with it, are predisposed to anemia, to syncope, and to other nervous affections. In some cases, however, we observe shortness of breath on the least exertion, palpitations without energetic contractions, discoloration of the integuments, and a precarious and sickly existence, œdema, and death, in a state of exhaustion.

"The treatment of atrophy is governed by the exciting causes."

We find it impracticable in the limits at our disposal, to give any satisfactory account of the remaining chapters of this practical little monograph, which, however, we deem of the less consequence, since all who are not familiar with the subject, and are beginning to feel an interest in its investigation, are pretty sure to possess themselves of the work. We close our notice of it with one extract more, relating to a cardiac affection not so formidable as many others, but distressing in its nature, and more frequent in its occurrence than any other malady of that important organ. We allude to *palpitations:*

"Palpitations consist in an increase of the force and frequency of the pulsations of the heart, so as to render them perceptible, and even disagreeable to the patient. They vary in force, from a degree in which they are hardly sensible to one of great violence. The patients often hear the pulsations, especially when they lie on the left side; even the two sounds of the heart may be distinguished in this posture. During the palpitations, the sounds of the heart ordinarily increase in intensity; and, in some circumstances, a slight bellows sound is produced, which ceases and disappears when the circulation has regained its natural quietness. Palpitations are also accompanied by a slight peripheric friction sound, which is dry and superficial, and due to the rapidity with which the two folds of the pericardium glide on each other: it disappears with the cause which originated it. At the same time, the arterial diastoles are short and quick, and the veins of the lateral parts of the neck are swelled and distended. Palpitations, when prolonged, always cause a sensation of uneasiness and anxiety in the præcordial region, and of fatigue in the whole extent of the chest; also, quickness of breathing, sometimes even extending to syncope.

"Palpitations form two great classes; viz: those which are caused by a material alteration of the heart, (*palpitations from organic cause*), and those which arise from a nervous cause (*palpitations from inorganic cause*), and are not referrible to any material lesion of this organ. *Palpitations from organic*

*cause* are distinguished from the others by their permanence and tenacity, by the great irregularity which they cause in the pulsations of the heart, and in the sounds, which are considerably feebler than common, and, finally, by the existence of the signs peculiar to the affection of the heart which has produced them.

"*Nervous palpitations*, of which only we intend to speak here, constitute a very troublesome disease. They present many varieties which it is important to distinguish, because their treatment is different, and sometimes even opposite. Everything else being equal, they are more violent in proportion as the patients have a more nervous constitution or a more irritable temper.

"In the *first variety* we shall place palpitations induced by strong mental emotions, by hysteria, hypochondriasis, nostalgia, excessive labour, particularly in the night, venereal excess, dyspnœa, &c. These are nervous *palpitations, properly so called.*

"In the *second variety* we place palpitations produced by the abuse of spirituous drinks, and by exciting regimen, and especially by plethora (*plethoric palpitations*).

The *third variety* will consist of palpitations which appear in individuals who are anemic and chlorotic, and which are owing to an impoverishment of the blood (*anemic palpitations*). These last palpitations are very often confounded with palpitations from organic causes, although it is easy to distinguish them by a close examination; for all the characters of anemia and chlorosis are present. We assure ourselves that the heart has not increased in size, that its orifices are in their natural state, and that the valves are completely closed. In speaking of the intra-cardiac murmurs, we have already given the means of distinguishing murmurs by organic causes from the murmurs by inorganic causes. We shall not repeat them here. The case most difficult to be recognized would be that where palpitations from organic and inorganic cause existed at the same time.

"*Progress, duration, termination.*—The progress of palpitations is always irregular; they ordinarily return in paroxysms at more or less distant intervals. As to their duration, they vary according to a number of circumstances, and especially according to the cause which has produced them. It is difficult to fix a termination to so transient an affection. It may, however, be said, that when they are prolonged, or are repeated for a long time, they finish by causing hypertrophy or dilatation of the heart.

"*Treatment.*—The treatment of palpitations is entirely subordinate to the causes which have produced them; and to the ascertaining of these must the efforts of the physician be directed. Palpitations from moral causes can only be managed by moral treatment. This last is not so successful against palpitations produced by hysteria or nostalgia. As to those which are produced by dyspepsia, the means which are commonly directed against this affection of the digestive canal should be employed. Plethoric palpitations require a more active treatment. Sanguineous depletions, moderate regimen, exercise, are the means which succeed the best. In anemic palpitations, tonics should be had recourse to, as well as the chalybeate preparations, cold baths, nourishing food, moderate exercise. The effects of chalybeates may be considered as the test of these palpitations. If they do not succeed, an organic affection is to be feared. The preparations and mode of administration must be varied. Among those of iron, we place, in the first rank, the subcarbonate and lactate of iron."

